# Are Doctors Becoming Unpopular?

**Published:** 1922-05

**Authors:** R. W. Harris

**Affiliations:** (formerly Assistant Secretary, responsible for the administration of National Health Insurance Medical Benefits, Ministry of Health)


### ARE DOCTORS BECOMING UNPOPULAR ?

**By R. W. HARRIS (formerly Assistant Secretary, responsible for the administration of National Health Insurance Medical Benefits, Ministry of Health).**

THIS heading, in a slightly different form, appeared over a leading article in "The Times," of April 4, and I have ventured to adopt it in pursuing my inquiry whether the panel doctor is "doing his job." "The Times" article contains much of the kind of loose criticism to which I have referred. We have been, says the writer, the victims of an experiment in medical socialism, and the Ministry of Health, as the successor of the Local Government Board, having no therapeutic tradition, has tightened the bonds in which the unfortunate insurance practitioner is caught. The criticism in this case is of the system rather than of the panel doctor, but it is impliedthat, under such a system, the panel doctor cannot avoid becoming unpopular. Let us, therefore, look at the matters to which reference is made by way of illustration of the tightening of the bonds. This, says "The Times," is what has happened since the Ministry of Health was set up. We have had a series of measures which include the appointment of Regional Medical Officers, the setting up of State Clinics, the institution of Record Cards of illnesses, and even Courts of Inquiry into the conduct of panel doctors. Now, the simple facts with regard to these four things are :—

(1) That provision was made in the Parliamentary Estimates, and money was voted by Parliament for Regional Medical Officers, in 1914—many years, that is, before the Ministry of Health came into existence ;

(2) no State Clinics have been set up ;

(3) Record Cards of illnesses were required at the outset to be kept by insurance doctors as one of the original conditions of the special Exchequer grant made to them in 1912 ; and

(4) Courts of Inquiry into the conduct of panel doctors have not only been in existence since the passing of the National Insurance Act of 1911, but were expressly required by that Act to be set up, and are designed solely for the protection of the doctors against unfair removal from the service.

I am not concerned to express an opinion on the policy of the Ministry of Health, but it is germane to my subject to suggest that if critics of the insurance system are to throw discredit on the medical profession, it is at least to be expected that their criticism should be based on a more carefully considered review of the facts. We are told that the public, suspecting that its erstwhile best friend is no longer untrammelled, has become suspicious. But surely the public will form their opinion as to the merits or demerits of the service from their own observation and experience of the day-to-day work of the panel doctor, rather than from the existence of rules and regulations about which they know little and care nothing.

What is the scope of the insurance doctor's duties ? Is the pay which he receives calculated to deter him from their effective performance ? Should he be allowed to take on his list an unlimited number of patients ?

The treatment which the insurance practitioner is required to give to his patients is defined by regulation as "such treatment as is of a kind which can consistently with the best interests of the patient be properly undertaken by a general practitioner of ordinary professional competence and skill," but it does not include treatment in respect of a confinement. This definition, it will be seen, does not extend to any specialist service which an individual practitioner, by reason of the possession of special skill, may be capable of rendering. Nor is the doctor in a position to send his patient, free of charge, to a specialist, or to a hospital, or to secure for him the services of a nurse except, of course, where the same service could be obtained gratuitously, through machinery independent of the Insurance Acts, for uninsured people. Such limitations on the service are imposed mainly by financial considerations.

The panel doctor fails in so far as he does not give wholeheartedly to his insurance patients the service which he would give within the above limits to his private patients. To the extent to which there is a falling short of this standard, one must look in part to the difficulty which doctors doubtless still experience in settling down under such an enormous change as that effected by the National Insurance Act. Human nature being what it is, it must be expected that doctors miss any direct and visible connection between the contribution of the insured person and his employer and the service which the doctor is called upon to give in return. There is also a tendency in certain quarters to foster in the minds of insurance doctors the idea that they are receiving a niggardly rate of remuneration. A doctor, recently writing to the Press, says that "the ordinary medical man of to-day is worth at least £2,000 a year." Well, it is an imperfect world, and medical men, as is the case with other poor mortals, have sometimes to give their best work for less than its full value. And as, in a service which every medical man has a right to enter, a flat rate of payment is inevitable, this means that it suffers from the inherent defect of being payable alike to good, bad, and indifferent doctors.

The fact that a 9s. 6d. capitation fee is payable to insurance doctors at the present time does not convey much meaning to the mind. A certain doctor, who hung up in his surgery a notice to the effect that he was receiving the shameful rate of about a farthing a day for this work, lived to regret his action, and to take down the notice. His arithmetic was not unreliable, but he managed to convey a wrong impression. Statistics show that an insurance doctor with 1,000 persons, well and ill, on his list—and the great majority have a list under 1,000—is called upon to give, on average, about ten or eleven surgery attendances a day, including those for minor ailments or renewals of certificates, and to pay four visits. This enables one to see in its true perspective the quantity of work involved, and also to calculate that for the portion of the day necessarily occupied by this work, and the incidental correspondence and record-keeping, the doctor is paid *at the rate* of about £1,200 a year net, after allowing a deduction for working expenses. That is for a town practice ; the position is different in the country, where the doctor receives a special payment for mileage.

If, therefore, a town doctor with 1,000 insured persons on his list devoted the rest of a working day to private practice, for which he received payment at the same rate, he would be earning £1,200 a year net. But a doctor, whether he has 1,000, 2,000, or 3,000 insured persons on his list, is not restricted as to the number of private patients to whom he may give treatment. And his actual income, therefore, is governed to a large extent by the number of insured persons for whom he chooses to accept responsibility, and the amount of time left available for private practice.

This leads me to the question of the effectiveness of imposing an arbitrary limit on the number of insured persons that a doctor may accept. The present outside limit is 3,000. In many counties and county boroughs it has been fixed locally at 2,000 or 2,500. At a recent conference of Approved Societies a resolution was passed calling for a lower universal maximum than 3,000. In my view experience shows that any general limit of this kind fails in its object. Within the limit, an incompetent practitioner, or one with too much private practice, or without adequate assistance, will give an unsatisfactory service. Another doctor, energetic, capable, and popular, will turn away insured persons who want to come to him for treatment. There should, in my view, be not a general limit for the country or for the county ; but a power to the Insurance Committee (with right of appeal to the Minister) to fix a limit for a particular district, doctor, or group of doctors, where experience shows it to be necessary.

The doctors have recently become disturbed because at the end of 1923, when the capitation fee falls due for reconsideration, the Approved Societies will be called into consultation on the question. I do not think that amongst those best qualified to speak for the insured persons there will ever be a desire to underpay the doctor, or to risk a " cheap and nasty" service. If it should appear from what I have written that there is any disposition on my part unduly to find excuses for the panel doctor, let me adjust this by a general reflection. As there is an average rate of remuneration, so there is an average quality of work, and for those who help to bring down the average there is a substantial period between now and the end of 1923 in which to convince the insured population that the medical service under the Insurance Acts is a good service, and one, therefore, for which it is worth while to pay well. Auto-suggestion is rather in evidence just now ; and there are some doctors who might say to themselves at night :—" I am receiving a fair rate of remuneration for my insurance work ; I have no bad debts ; and no troubles of collection." And in the morning " I am giving a better and better service every day."

